# Acceptance and commitment therapy for adults with advanced cancer (CanACT): study protocol for a feasibility randomised controlled trial

**DOI:** 10.1186/s13063-016-1169-8

**Published:** 2016-02-11

**Authors:** Joseph Low, Marc Serfaty, Sarah Davis, Victoria Vickerstaff, Anna Gola, Rumana Z. Omar, Michael King, Adrian Tookman, Janet St John Austen, Karen Turner, Louise Jones

**Affiliations:** Marie Curie Palliative Care Research Department, Division of Psychiatry, University College London, 6th Floor, Maple House, 149 Tottenham Court Road, London, W1T 7NF UK; Division of Psychiatry, University College London, 6th Floor, Maple House, 149 Tottenham Court Road, London, W1T 7NF UK; Department of Statistical Science, University College London, 1-19 Torrington Place, London, WC1E 7HB UK; Marie Curie Hospice Hampstead, 11 Lyndhurst Gardens, London, NW3 5NS UK; PPI Representative, London, UK

**Keywords:** Feasibility, Randomised control trial, psychological therapy, Acceptance and commitment therapy, Advanced cancer

## Abstract

**Background:**

One-third of people with cancer experience psychological distress and may suppress distressing thoughts, emotions, and concerns, leading to further problems. Conventional psychological treatments reduce distress by problem solving, but in advanced cancer, when ill health is progressive and death may be approaching, physical and psychological difficulties are complex and have no simple solutions. Acceptance and Commitment Therapy encourages acknowledgement and acceptance of mental experiences, increasing people’s ability to work with problems that cannot be solved. Previous pilot work in advanced cancer confirms that distress can be associated with an avoidance of experiencing uncomfortable thoughts and emotions.

**Methods/Design:**

This feasibility randomised controlled trial of Acceptance Commitment Therapy aims to establish parameters for a larger trial. Fifty-four participants with advanced cancer will be randomly allocated to up to eight sessions (each 1 hour) of Acceptance Commitment Therapy or a talking control. Participants will be recruited from those attending outpatient services and hospice day care at three specialist palliative care units in North and East London, United Kingdom. The primary outcome is a measure of functioning in four areas of life (physical, social/family, emotional, and general activity) using the Functional Assessment of Cancer Therapies - General questionnaire at 3 months after randomisation. Secondary outcomes are (i) acceptance using the Acceptance and Action Questionnaire; (ii) psychological distress using the Kessler Psychological Distress Scale; (iii) physical functioning using a timed walk and sit-to-stand test; and (iv) quality of life measures including the Euroqol-5 Dimensions and ICECAP Supportive Care measures. Qualitative data will be collected at 3 months to explore the participants’ experiences of the trial and therapy. Data will be collected on the costs of care.

**Discussion:**

Data generated on the recruitment, retention, and experience of the interventions and the usefulness of the outcome measures will inform the adaptations required and whether changes in function are consistent with existing data when planning for a sufficiently powered randomised controlled trial.

**Trial registration:**

ISRCTN13841211 (registered 22 July 2015).

**Electronic supplementary material:**

The online version of this article (doi:10.1186/s13063-016-1169-8) contains supplementary material, which is available to authorized users.

## Background

Patients with advanced cancer experience deterioration in both physical and psychological functioning as their disease progresses [35]. Palliative care aims to improve the quality of life in the face of increasing symptom burden and disability [36]. Psychosocial interventions may be useful in achieving improved quality of life [33] and the National Institute of Health and Care Excellence recommends psychological interventions [29] in the treatment of psychological distress in people with cancer. However, the intrusion of negative thoughts or feelings associated with a deteriorating disease that may no longer be amenable to cure often reduces the ability to address any social, psychological, or spiritual problems [41]. New approaches are required to support people to develop strategies to manage their negative emotions more effectively, especially when dealing with life-threatening illness when problems often do not have specific solutions.

Acceptance and Commitment Therapy (ACT) is a third wave cognitive behavioural therapy (CBT). It is novel as it encourages individuals to be willing to experience and manage negative emotions and thoughts. Unlike conventional CBT, ACT encourages psychological flexibility, which enables people to manage their distress. Indeed, avoidance of uncomfortable feelings (experiential avoidance) may maintain psychological suffering [16]. Typically delivered as a one-to-one process with a therapist, ACT targets six core psychopathological processes by addressing acceptance, cognitive diffusion, self as context, values, and being present and committed action [18]. ACT may be particularly relevant in advanced cancer as it aims to encourage patients to tolerate problems and direct their behaviours towards living in the present moment rather than focussing on their fears for the future [20]. This may be useful for those facing a limited prognosis and for whom death is approaching. ACT does not encourage acceptance of pain or discomfort that can be ameliorated by effective medical treatments; rather it is about accepting the inevitability of discomfort and physical limitation that can occur in cancer and preventing the capacity for suffering.

ACT has been evaluated in a number of health-related behavioural interventions [11, 14, 27, 42], and participants receiving ACT are more likely to report better physical and psycho-social outcomes compared to those receiving treatment as usual [32]. A recent review has identified six studies using ACT in cancer settings, which have provided useful pilot data but have been methodologically limited, both in sample size and the reporting of cancer stages [20]. No published studies have evaluated the impact of ACT on improving both psychological and physical functioning in patients specifically with advanced cancer.

We began with preliminary work to understand the relevance of ACT in advanced cancer. We conducted a cross-sectional study of 101 people with advanced cancer attending a specialist palliative care outpatient unit to explore the relationship between experiential acceptance (a preparedness to experience unpleasant or unwelcome thoughts and emotions without struggling to solve or resist them) and both psychological morbidity and physical function [24]. We found that those with high levels of experiential acceptance were less likely to report psychological distress and have improved physical function. This suggests that ACT therapy to encourage experiential acceptance may be of benefit in this population.

We now plan a phase II randomised controlled trial (RCT) to determine the feasibility and acceptability of delivering an ACT intervention with people with advanced cancer and obtain data to inform the conduct of a future larger, definitive trial. We propose a two-arm trial, which we have called CanACT, to compare ACT to a talking control, and in which treatment as usual will be available to all participants. The talking control intervention (TC; [38]) has been developed to control for the main non-specific factors that may operate in psychological talking-based therapy. Expressing feelings in a therapeutic environment, rather than any specific aspect of ACT, may be sufficient for improvement.

Although economic analyses in psycho-oncology are infrequent [6], there is evidence to suggest psychologically based interventions lead to lower utilisation of healthcare resources in cancer generally [34, 39, 43] and in metastatic breast cancer [22].

In this paper, we report the protocol (version 8, 21 August 2015) for a two-arm feasibility randomised controlled trial of the clinical and cost effectiveness of ACT plus treatment as usual (TAU) compared to a talking control (TC) plus TAU for people with advanced cancer attending hospice ambulatory services.

We shall undertake the following tasks in this study:

1. Test the feasibility of recruitment to the trial, retention and attrition.
2. Explore the feasibility of providing a therapist-delivered intervention in this setting.
3. Assess the usefulness and acceptability of a range of clinical and economic outcomes.
4. Assess if the data generated from the outcomes in this trial support the conduct of a larger RCT.
5. Use qualitative methods to understand participants’ perceptions of whether ACT and TC are feasible and acceptable interventions.

## Methods/Design

### Design

This is an exploratory, two parallel arm, randomised controlled trial with a nested qualitative study.

### Setting

Recruitment of participants and implementation of the intervention will take place in three specialist palliative care day therapy units in North and East London, United Kingdom (UK). Each unit serves diverse populations with a broad mix of ethnic and socio-economic backgrounds representative of an inner city. All offer hospice day care services consisting of a mixture of medical-led, nurse-led and allied health professional-led care. People may begin accessing these services either at the end of active cancer treatments, during palliative cancer treatments, or for symptom control in the advanced stages of their illness.

### Support, funding and ethics

This trial is supported by the UCL PRIMENT Clinical Trials Unit (https://www.ucl.ac.uk/priment). Funding for the trial is provided by the National Institute for Health Research (UK) through their Research for Patient Benefit funding stream. Ethical approval has been obtained from the London - Riverside Research Ethics Committee (14/LO/0813). Local permission to conduct the study was given by the following organisations: Marie Curie Hospice Hampstead, St Joseph Hospice Hackney and the Hospital of St John and St Elizabeth Ethics Committee. All relevant parties will be informed about any important modifications to the protocol. The SPIRIT guidelines for this protocol are attached in the Additional file 1, highlighting which recommended items we have addressed in the reporting of this protocol. 

### Quantitative arm

#### Participants

We shall recruit 54 patients, with 27 in each arm. We chose this number on pragmatic grounds because it is sufficient to demonstrate feasibility in terms of recruitment, acceptance of randomisation, and likely attrition.

#### Inclusion criteria

Eligible patients will meet the following criteria: i) aged > 18 years; ii) a clinical diagnosis of advanced cancer that is not amenable to curative treatment (that is, those with metastases at diagnosis, those at first or subsequent extensive recurrence, and those receiving palliative treatments) and iii) exhibit a total score of < 81 on the Functional Assessment of Cancer Therapies - General (FACT-G). This score was selected as the mean score of the FACT-G in a cancer population is 80.9 (SD 17.0) [5] and depicts an important degree of dysfunction.

#### Exclusion criteria

Patients will be excluded if i) the clinician estimation of patient survival is < 4 months; ii) the patient has insufficient command of English to engage in ACT or participate in talking control; iii) the clinical or the research team considers the patient to be cognitively impaired; or (iv) the patient is currently receiving CBT (including ACT).

### Recruitment process

The research team, together with clinic staff, will review patient lists on a weekly basis. Clinicians will ask patients identified as eligible to give initial consent to be involved in the study. The research team will then complete the FACT-G with patients who gave initial consent. A researcher will give those scoring below the threshold of 81, verbal and written information about the study. These patients will be given 48 hours to consider the study, after which written informed consent for trial entry will be obtained, baseline assessments will be collected, and randomisation will occur.

### Randomisation

Participants will be randomised to either ACT or the TC group. Randomisation will be 1:1 to each treatment arm and will be stratified by centre. Block randomisation with varying block sizes will be used to recruit equal numbers to each arm. The randomisation list is being generated by PRIMENT CTU. The randomisation will be performed by a senior member of the research team not connected with either patient recruitment or data collection, who will inform the therapists of the participants’ allocation. The participants, the researcher collecting the data from trial participants, and the trial statisticians will be masked to the randomisation.

### Interventions

There are two intervention arms: Acceptance Commitment Therapy (ACT) and Talking Control (TC). For both interventions, up to eight sessions (each 1 hour) will be offered weekly and delivered within 3 months. Sessions will usually take place face to face in a palliative care day therapy unit but the final three may occur at home if requested [37].

#### Acceptance Commitment Therapy

ACT is a contextual behavioural approach that uses a collection of techniques aimed at increasing psychological flexibility to change outcomes. Psychological flexibility is the ability to persist in valued life activities alongside distressing or unwanted private events. There are two main processes (i) mindfulness and acceptance (cognitive fusion and self as context) and (ii) commitment and behaviour change (being present, defining valued directions, and committed action). We shall model the therapy on the work of Hayes et al. [18] and supplement the approach with the manual ‘Get out of your mind and into your life’ [17]. Therapists will be familiar with the skills training manual ‘Learning act’ by Luoma et al. [27] and ‘ACT made simple’ by Harris [15] and have received training from the Chief Investigator.

#### Talking control

TC promotes the use of common factors in therapy by encouraging the therapist to be warm and welcoming, allowing people to express their feelings, feel heard, and understood. It specifically discourages focusing on problem areas and stipulates that problem solving should not be attempted. The therapists are trained to adhere to guidelines, and this approach has been used previously to control for common factors in therapy [38].

### Procedure

We shall collect baseline assessment measures from patients before they are randomised to either the ACT or TC group. A senior member of the research team will notify the therapists of the group allocation of participants. The therapists will contact participants directly to arrange therapeutic sessions. The research associate will approach all participants face to face either in hospice outpatient settings or in their homes (their preference) to collect follow-up data to a maximum of 6 months post-baseline. At each time point, we will offer each participant up to three opportunities to do the follow-ups. We shall also offer follow-up by telephone if necessary to minimise attrition and missing data, as participants may become increasingly unwell during the study. A research associate specifically employed for this study will conduct the assessments. Table 1 summarises the assessment schedule. Although participants and therapists cannot remain masked to treatment group, the research team (with exception of the research nurse and the senior researcher – see qualitative arm) will be masked. As a check on blindness, research associates and will be asked to guess group allocation after they have completed each final follow-up assessment. Unmasking will not occur until databases are closed and the main analysis has been completed.

**Table 1:** Timing of data collection

Measures	Baseline	1 ½ months post baseline	Post-intervention (3 months)	4 ½ months post baseline	Follow-up (6 months)
Functional Assessment of Cancer Therapy - General	✓	✓	✓	✓	✓
Kessler Psychological Distress Scale	✓		✓		✓
2 minute walking test	✓		✓		✓
1 minute sit to stand test	✓		✓		✓
Acceptance and Action Questionnaire-II	✓		✓		✓
Value Living Questionnaire	✓		✓		✓
EQ5-D	✓		✓		✓
ICECAP supportive care measure	✓		✓		✓
Client Service Receipt Inventory	✓		✓		✓
Satisfaction with care			✓		
Counselling questionnaire			✓		
Expectation of therapy	✓				
Treatment preference	✓				
Assessment of blindness			✓		✓
Attrition			✓		✓
Other therapies used			✓		✓

#### Treatment fidelity

There will be five therapists delivering either one of the two interventions across the three sites. The same therapist will deliver both ACT and TC to reduce group differences in non-specific factors (for example, warmth and professionalism). Therapists will have a minimum of two years’ experience in delivery of psychotherapy and will be accredited with the British Association of Cognitive Behavioural Psychotherapists. To standardise the two interventions and to ensure that the two therapies are kept strictly separate, all therapists will receive a training manual detailing both interventions and 1.5 days of training provided by the Chief Investigator (MS). To ensure treatment fidelity, a random sample of 1 in 10 audio-taped sessions will be selected and stratified according to therapist and phase of the intervention (early, mid or end) and rated for quality using: a) ACT - an adapted form of the scale developed by Plumb and Vilardaga [31] to assess treatment integrity and b) TC - a modified checklist [38] for supervision to check that TC is being delivered. The Chief Investigator will discuss issues relating to the delivery of therapy with any therapist who is not consistent in their delivery of ACT or TC.

### Measures

Data on all measures will be recorded at baseline, 3 months (post-intervention) and 6 months. The FACT-G will additionally be recorded at 1.5 months and 4.5 months to take into account the poor health of the patient group (15 % may die by the 6-month follow up [24]) and allow maximal inclusion of data from all participants for our main outcome measure. Demographic data will be recorded at baseline only.

#### Demographic data

The following will be recorded from each participant: age, gender, ethnicity, marital status, education, previous psychiatric history, type of cancer and when diagnosed, and the occupation of the main salaried person in the household before retirement.

#### Patient functioning

Functional Assessment of Cancer Therapies – General (FACT-G) (Version 4; [7]) is the main primary outcome, as its four wellbeing domains (physical, social/family, emotional, and activity) best reflect the main goals of ACT. With high internal consistency reliability and validity, it is recommended for assessing health-related quality of life in advanced cancer [25]. Pilot work has suggested that this measure is acceptable to use for our population.

#### Psychological distress

The Kessler Psychological Distress Scale (K10; [21]), a short self-report measure will be used to assess psychological distress. This measure was used in our preliminary study [25].

#### Physical functioning

Two measures will be used to evaluate physical functioning, as recommended by specialist physiotherapists working in cancer palliative care. These are the two minute walking test (that is, the distance walked in 2 minutes in a controlled space, at own speed, using an aid if necessary) and the 1 minute sit to stand test (that is, the number of times a person can stand up and sit down from a standardised chair over 1 minute [23, 30]). Both measures were used in our preliminary study (Low et al. [25]).

#### Process of ACT

Two measures will be used to evaluate two different processes of ACT. The Acceptance and Action Questionnaire II (AAQII; [2]) will measure experiential avoidance and participants’ willingness to accept undesirable thoughts and feelings, whilst acting in congruence with personal values and goals. This measure was used in our preliminary work [25]. The Valued Living Questionnaire (VLQ; [44]) will measure how consistent their actions are with the values they consider are important.

#### Economic

The following three economic measures will be used: i) the EQ-5D ([4, 12]), a commonly used generic utility measure of quality of life; ii) ICECAP supportive care measure (ICECAP-SCM; [9]), a seven item capability index specifically developed for assessment of people with supportive and palliative care needs; and iii) short modified version of the Client Services Receipt Inventory (CSRI, [1]) to collect service use of participant. We are using both the EQ-5D and ICECAP-SCM in order to determine the most appropriate health-related quality of life measure for use in a larger trial.

#### Expectation, experience and satisfaction of therapy

At baseline, participants will be asked to assess their expectation of therapy using a single question [3], and their treatment preference, using two questions [37]. At 3 months, participants will be asked to assess their experience of therapy, using a shortened version of the Counselling Questionnaire [10], and a self-constructed five-item questionnaire to rate their satisfaction with therapy.

#### Assessment of blindness

The researcher undertaking the assessments will be asked to guess trial arm allocation for each participant after final follow-up.

#### Attrition

Dropout from therapy and reasons for not attending therapy sessions will be recorded, along with loss to follow-up (for example, dislike of therapy, deteriorating health, and death).

#### Other therapies used

Participants’ medical notes will be accessed to obtain the information on important covariates: (a) prescribed medications including the dose and changes in prescribed analgesic and psychotropic medication; (b) other psychological therapies offered as part of treatment as usual, which will include any psychological intervention received during the trial (for example, hypnotherapy, art therapy, counselling, CBT, spiritual healing, or relaxation); (c) complementary therapies received during the trial (for example, aromatherapy, massage, Reiki, reflexology, and herbal remedies); and (d) gym and physical therapies.

### Data analysis

Given this is a feasibility randomised trial, our main analysis will proceed as follows. We shall examine the numbers of eligible patients, the recruitment rate, loss to follow-up, and adherence to therapy in both arms descriptively. We shall determine, through completion rates, the appropriateness and acceptability of the outcome measures. Our primary outcome is the FACT-G score measured at 3 months follow-up because, at this point, we anticipate that participants will have completed their therapy, and the majority will remain well enough for full data collection. Patient characteristics will be summarised by trial arm using mean (SD), median (interquartile ranges) or proportions as appropriate. We shall estimate the intervention effect with 95 % confidence intervals using linear regression adjusted for baseline values of FACT-G score and centre or suitable alternatives in case of non-normally distributed data. This will inform the sample size calculation for a main trial. We will use regression models that account for clustering to examine how the repeated measures of FACT-G score vary over time. Analysis will be carried out on an intention to treat basis. We will use the feasibility study results to examine therapist clustering descriptively. We will also conduct a preliminary economic analysis.

We anticipate that 45 patients will complete the trial, assuming 15 % attrition [24]. We shall examine the characteristics of participants who drop out of each trial arm and the reasons for attrition when available. This will help us to understand the pattern of missing data and to make appropriate plans to handle these missing data in a larger scale RCT. All analyses will be performed using Stata 13 or above [40].

### Qualitative arm

A nested qualitative arm is incorporated into this trial for a richer description of the patient experience of receiving ACT or TC. A purposive sample of 20 participants at 3 months post-baseline will be invited to take part in qualitative interviews to understand experiences of therapy. In selecting participants, we shall to take into account the allocated treatment, severity of illness, gender, age, and ethnicity. The interviews, conducted by the research nurse, will explore what participants found helpful or unhelpful, any alterations in behaviour, the experience of working with the therapist, any factors that might inhibit the use of either ACT or TC and what might contribute to non-engagement with therapy, including potential reasons for withdrawal from therapy and from this study. Interviews will be transcribed verbatim and imported into NVIVO v10 (QSR) [29]. Two members of the research team (the research nurse and a researcher) will independently read the transcripts in-depth and using thematic content analysis [13] extract emerging themes. Any disagreements will be resolved by consensus. We shall explore how themes are linked and whether variables such as age, gender, ethnicity, severity of illness or perceptions of therapy exert an effect.

## Discussion

In this feasibility trial, we shall determine the feasibility of recruiting patients with advanced cancer to a randomised controlled trial of ACT versus TC in this setting. This will include follow-up rates, attrition and outcome measures to inform the design of a larger trial. Although we cannot at this stage test the effectiveness of this therapy, we shall learn whether it is acceptable to people attending hospice outpatient services. Our quantitative data will also provide an initial understanding of the potential for benefit of ACT for people facing advanced cancer and death. Our qualitative data will increase our understanding of the experience of receiving both ACT and TC sessions, in particular which elements of the interventions are most valued by participants.

Since participants are likely to experience clinical deterioration as their disease progresses during the study period, our trial is designed to allow flexible options for delivery of the therapies and for follow-up assessments. Participants will be contacted up to four times, either by telephone or at a day hospice appointment, to arrange the follow-up appointments. It will be important to retain as many participants in the trial for as long as possible, in particular because it may be that the impact of the ACT interventions become more relevant as health deteriorates, particularly as attempting to restore function through problem solving will not be possible.

We have incorporated health economic assessments, recognising that economic evaluation is an important component of any interventional study as providers of healthcare require an evidence base to ensure that resources are used wisely. Should this study demonstrate the feasibility of ACT as an intervention, we would proceed to a fully powered RCT, the design of which would be informed in part by the proposed value of information analysis [13, 16] . Value of information analysis is increasingly part of the standard methodology of health economists. Within a full trial, a comprehensive set of resource use data would be required to evaluate the cost-effectiveness of the intervention.

The CanACT study is the first controlled trial to evaluate individual ACT compared to a talking control, for treating people with advanced cancer and dysfunction. The mixed methods used will help generate preliminary guidelines on how ACT may best be delivered. If ACT appears beneficial, then this will help generate preliminary guidelines that support the use of ACT in an advanced cancer population. In the medium term, findings will indicate whether or not a fully powered RCT is feasible. If ACT proved to be cost-effective in such a trial, it could become one of the recommended treatments for palliative care patients. This could be delivered either through specialist palliative care services or through the IAPT programme, whose goal is to also treat longer term conditions [11], and which may be adapted to treat people with cancer. We will also disseminate the results of the study in international journals and conferences.

### Confidentiality

All study participants will be allocated a unique study ID number for all written and electronic study data. All study data will be fully anonymised and any personal identifiable demographic information will be kept separately from the research results. All electronic records will be password protected. All paper records will be securely locked in a filing cabinet after use. In protecting participants’ confidentiality (potential and enrolled), the research team will abide by the regulations set in the United Kingdom Data Protection Act 1998.

## Trial status

A Research Associate responsible for patient recruitment has been engaged since May 2015.

Patient recruitment will commence in October 2015.

## Additional file

Additional file 1:
**ACT protocol paper SPIRIT 2013 Checklist (2015 09 30).** File outlining how this protocol paper meets the different guidelines from the SPIRIT 2013 Checklist. (DOCX 22 kb)

## Abbreviations

AAQ-II: Acceptance and Action Questionnaire II
ACT: Acceptance and Commitment Therapy
CBT: Cognitive Behavioural Therapy
EQ-5D: Euroqol-5D
FACT-G: Functional Assessment of Cancer Therapies – General
K-10: Kessler Psychological Distress Scale
RCT: Randomised Control Trial
TAU: Treatment As Usual
TC: Talking control
VLQ: Valued Living Questionnaire
